# Visual analysis of research hotspots and emerging trends in the field of newly graduated nurses

**DOI:** 10.3389/fmed.2025.1641430

**Published:** 2025-12-15

**Authors:** Jiexuan Xu, Murong Lu, Xuemei Liu, Yu Zhai, Jianqin Huang, Yile Tian, Jiayan Liang, Shuting Liu, Hongjing Yu

**Affiliations:** The Second Affiliated Hospital of Guangzhou Medical University, Guangzhou, China

**Keywords:** newly graduated nurses, bibliometrics, visual analysis, research hotspots, emerging trends

## Abstract

**Aim:**

To systematically analyze the current status, hotspots, and frontiers of Chinese and international studies on newly graduated nurses and to inform subsequent research.

**Methods:**

We searched the Web of Science Core Collection and China National Knowledge Infrastructure for literature on newly graduated nurses from January 1, 2015, to April 28, 2025, and used VOSviewer and CiteSpace to visualize and analyze the results.

**Results:**

In total, 4,372 English-language and 3,218 Chinese-language articles were included. English-language publications increased steadily, whereas Chinese-language publications showed a fluctuating decline. Both Chinese and English literature focus on mental health issues, with English literature focusing on nursing education and professional experience, and Chinese literature focusing on pre-service training and specialty competence development; English-language studies emphasize clinical decision-making, pre-entry interventions, and related frontiers, whereas Chinese studies further explore transition-shock mechanisms and localized support systems.

**Conclusion:**

In research on newly graduated nurses, Chinese- and English-language literature shows distinct focal areas. Future work should enhance international exchange and interdisciplinary collaboration, optimize digitally enabled training pathways, promote deeper integration of practice and education, and establish support systems for the professional development of newly graduated nurses that balance local and global perspectives.

## 1 Introduction

With the development of the global economy and healthcare, the social demand for nursing professionals is increasing (1). According to the World Health Organization, as of 2023, 40% of nursing staff are projected to retire, creating a global need for approximately 12.9 million newly graduated nurses to maintain clinical stability (2). As new members of the nursing workforce, newly graduated nurses substantially influence overall care quality and patient satisfaction through their professionalism, skills, and psychological resilience (3, 4). However, newly graduated nurses frequently experience role-transition conflicts, burnout, and high turnover, jeopardizing the stability of nursing teams worldwide (5, 6).

Addressing these challenges requires acknowledging that their manifestation and resolution vary substantially across healthcare contexts. As the country producing the world’s largest annual cohort of nursing graduates and operating a uniquely high-volume healthcare model, China offers invaluable empirical insights into scalable training systems and transition challenges under distinct pressures (7, 8). Conversely, English-language scholarship—predominantly from Western contexts such as the United States, the United Kingdom, and Australia—reflects different healthcare infrastructures, educational models, and policy frameworks. Analyzing these two substantial yet contrasting bodies of knowledge enables the identification of both shared transition challenges faced by newly graduated nurses globally and context-specific innovations often invisible within single-context studies (9).

Driven by these pressing needs and contextual diversity, research on newly graduated nurses has expanded dramatically over the past decade. We strategically selected the study period from 2015 to 2025 as it encompasses pivotal developments such as China’s 2016 national standardized training mandate for newly graduated nurses and the unprecedented global workforce strains amplified by the COVID-19 pandemic (9, 10). This timeframe allows for robust tracking of research evolution and meaningful comparison of thematic trajectories. However, this burgeoning, linguistically divided, and contextually dispersed body of literature has created a significant synthesis challenge, overwhelming the capabilities of traditional narrative review methods (11).

Consequently, a critical knowledge gap persists: there is a lack of systematic, comparative insight into how research priorities and thematic developments have diverged or converged between the Chinese and international literature, and how contextual factors have shaped these trajectories. This gap obscures cross-contextual patterns and limits opportunities for mutual learning and the effective transfer of evidence-based strategies.

To address this gap and objectively analyze the voluminous literature, we employed bibliometric analysis. This method is uniquely suited for mapping the knowledge structure and evolution of a research field by quantifying large-scale publication patterns (12), and its efficacy in identifying research trends and collaborative networks within healthcare is well-established (13). In this study, we apply bibliometric techniques to analyze both English and Chinese literature on newly graduated nurses published over the past decade. By visualizing thematic clusters, tracing temporal shifts, and profiling influential authors, institutions, and collaboration networks, we aim to construct a comprehensive comparative map of the global newly graduated nurses research landscape. This knowledge foundation will inform future research directions, strengthen targeted interventions, and ultimately support workforce retention and patient care quality worldwide.

## 2 Methods

### 2.1 Data sources and search strategy

The English literature search platform was the Web of Science Core Collection (WoS), the search formula was TS = “new nurs*” OR TS = “novice nurs*” OR TS = “newly graduated nurs*” OR TS = “junior nurs*” OR TS = “newly registered nurs*” OR TS = “newly qualified nurs*” OR TS = “newly qualified nurse*” OR TS = “neophyte nurse*.” The Chinese literature search platform was China National Knowledge Infrastructure (CNKI), and the search formula was SU = new nurse + newly graduated nurse + newly recruited nurse + low seniority nurses. All searches were restricted to January 1, 2015–April 28, 2025

### 2.2 Inclusion and exclusion criteria

**FIGURE 1:**
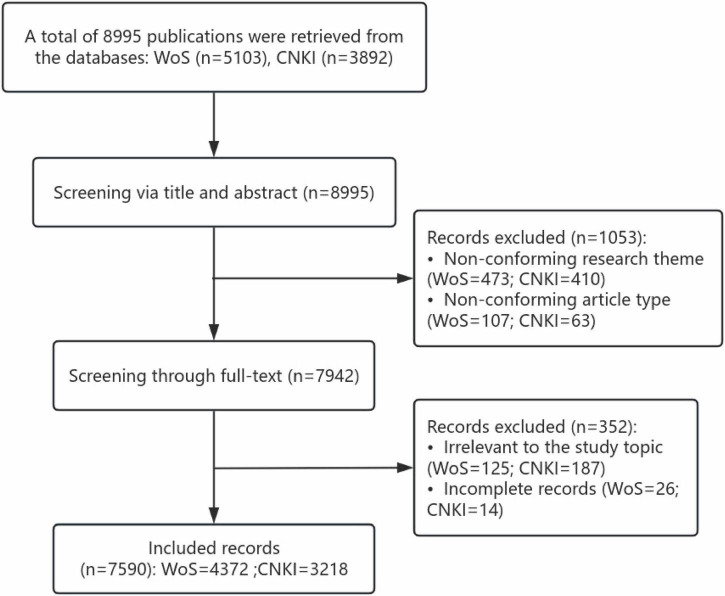
Literature screening process and results.

Inclusion criteria required articles focusing on newly graduated nurses, specifically original research or review articles published in academic journals. Exclusion criteria encompassed conference papers, letters, commentaries, calls for papers, and other non-article materials. The screening procedure involved two phases. First, articles were excluded based on titles/abstracts if irrelevant or incomplete. Then, the remaining articles underwent full-text evaluation against predetermined criteria. To ensure objectivity, two researchers performed independent screening, with any discrepancies addressed through consensus discussions or adjudication by a third researcher. The literature screening process is illustrated in Figure 1.

### 2.3 Research tools and data processing methods

We used VOSviewer 1.6.20 and CiteSpace 6.3.1 to perform co-occurrence and co-citation analyses of countries/regions, journals, authors, institutions, and keywords. In VOSviewer, co-authorship and keyword co-occurrence analyses were performed with the full counting method and a minimum keyword occurrence threshold of 15. Synonym merging was applied via a thesaurus file to ensure terminological consistency. In CiteSpace, keyword clustering and burst detection analyses were configured with time slicing from 2015 to 2025 (1-year per slice), using the g-index (*k* = 25) for term selection and the pathfinder algorithm for network pruning. The robustness of the resulting clusters was quantitatively validated using modularity (*Q* > 0.3) and silhouette (*S* > 0.5) scores (14). Finally, network visualizations and temporal trends from both tools were collectively interpreted to delineate the research landscape.

## 3 Results

### 3.1 Number of published studies

**FIGURE 2:**
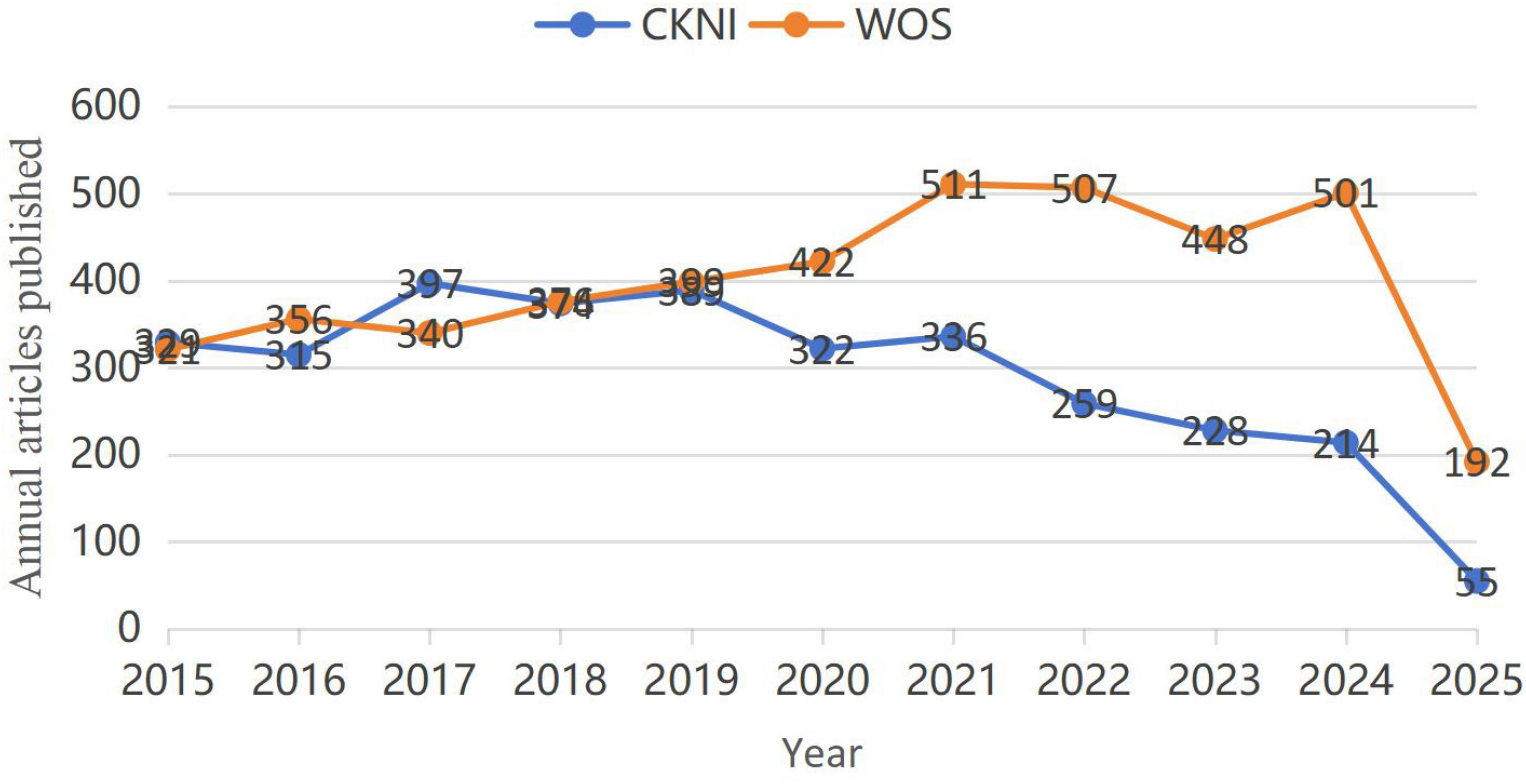
Annual publications in the field of newly graduated nurses from 2015 to 2025.

A total of 5103 relevant articles were retrieved from the WoS database, and 3892 relevant articles were retrieved from the CNKI database. After excluding non-compliant literature, 4372 English articles and 3218 Chinese articles were finally included. Visualizing and comparing the amount of Chinese and English literature published in the field of new nurse research between 2015 and April 2025 reveals a significant difference in the distribution of time between the two databases, CNKI and WoS: the English literature has been steadily increasing since 2015, with the increase being especially noticeable after 2020, while the Chinese literature exhibits a fluctuating upward trend, reaching a stage peak between 2018 and 2020, and then gradually declining from 2021 onward, as shown in Figure 2.

### 3.2 Visual analysis of countries and institutions

The global English literature publication volume of the top 10 countries in the field of new nurse research is shown in Table 1, with the United States leading at 36.43% and a centrality index of 0.40. The top five Chinese and international institutions in terms of publication volume are listed in Table 2. Internationally, research institutions from Australia and the United States dominate the leading positions, while in China, Huazhong University of Science and Technology and its affiliated hospitals are primarily represented.

**TABLE 1:** Top 10 countries in terms of English language literature publications in the field of newly graduated nurses from 2015 to 2025.

Country	Number of publications	Percentage(%)
United States	1593	36.43
Australia	619	14.15
China	424	9.69
United Kingdom	307	7.02
Canada	245	5.61
New Zealand	213	4.87
South Korea	151	3.45
Sweden	142	3.25
Taiwan, China	117	2.68
Spain	106	2.43

**TABLE 2:** Top 5 Chinese and international institutions in terms of publications in the field of newly graduated nurses from 2015 to 2025.

Classification	Institutions	Number of publications
International	Monash University	96
	University System of Ohio	85
	University of Sydney	72
	Harvard University	66
	University of Queensland	64
Chinese	Tongji Hospital, Tongji Medical College, Huazhong University of Science and Technology	15
	Union Hospital, Tongji Medical College, Huazhong University of Science and Technology	10
	Department of Nursing, Tongji Hospital, Tongji Medical College, Huazhong University of Science and Technology	8
	School of Nursing and Health, Zhengzhou University	8
	Xiangya School of Nursing, Central South University	8

### 3.3 Visual analysis of journals

Publications related to newly graduated nurses in English have been published in 920 journals, with Nurse Education Today contributing the most articles (*n* = 232), followed by Nurse Education in Practice (*n* = 171), Journal of Clinical Nursing (*n* = 132), Journal of Continuing Education in Nursing (*n* = 124), and Journal of Advanced Nursing (*n* = 117). In Chinese literature, studies on newly graduated nurses have appeared in 337 journals, with the top five journals by publication volume being Chinese General Practice Nursing (*n* = 129), Journal of Traditional Chinese Medicine Management (*n* = 119), the Journal of Nursing Science (*n* = 118), Practical Clinical Nursing Electronic Journal (*n* = 113), and Chinese Nursing Education (*n* = 101) (see Table 3 for details).

**TABLE 3:** Top 5 Chinese and international journals in terms of number of articles published in the field of newly graduated nurses from 2015 to 2025.

Classification	Journals	Number of publications
International	Nurse education today	232
	Nurse education in practice	171
	Journal of clinical nursing	132
	Journal of continuing education in nursing	124
	Journal of advanced Nursing	117
Chinese	Chinese General Practice Nursing	129
	Journal of Traditional Chinese Medicine Management	119
	Journal of Nursing Science	118
	Practical Clinical Nursing Electronic Journal	113
	Chinese Nursing Education	101

### 3.4 Visual analysis of author collaboration networks

**FIGURE 3:**
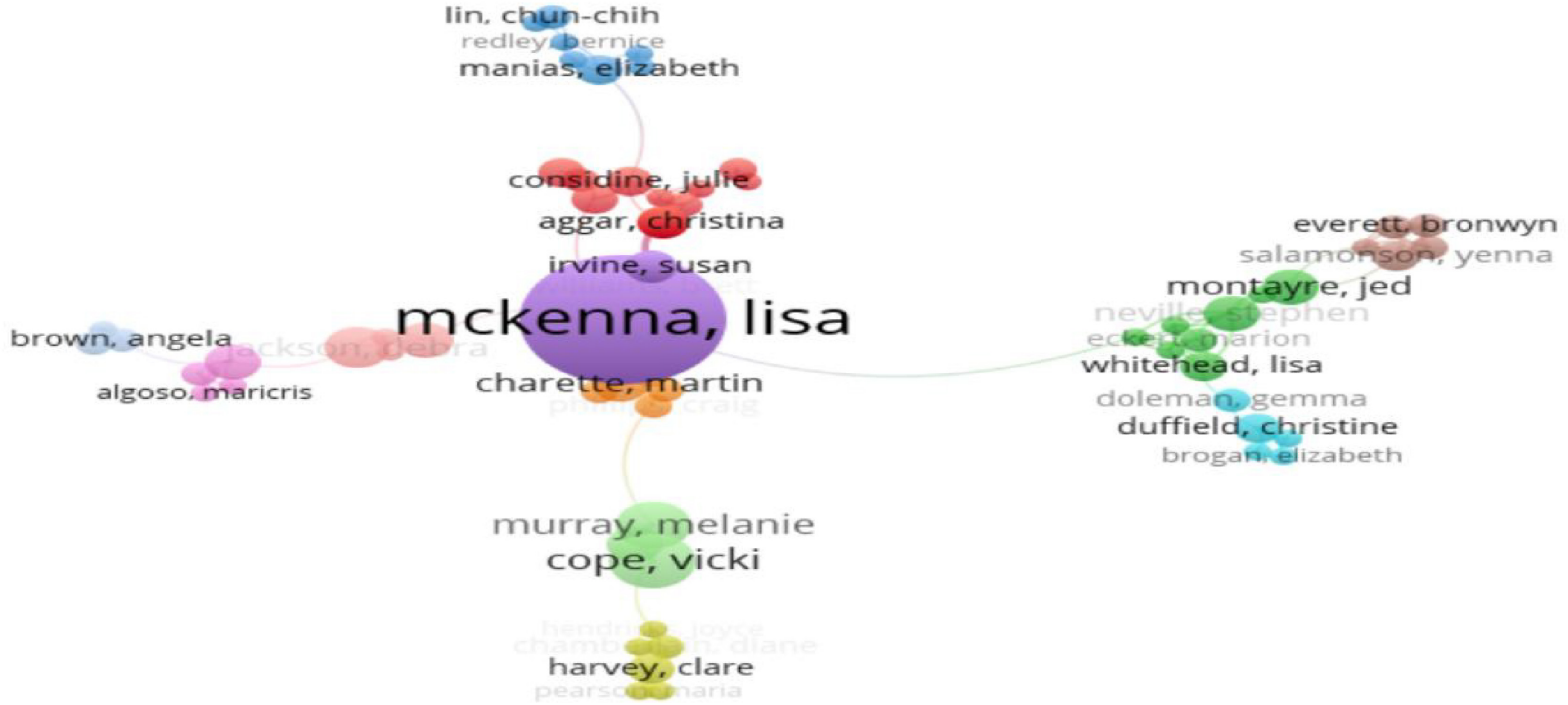
Collaborative network map of international authors.

**FIGURE 4:**
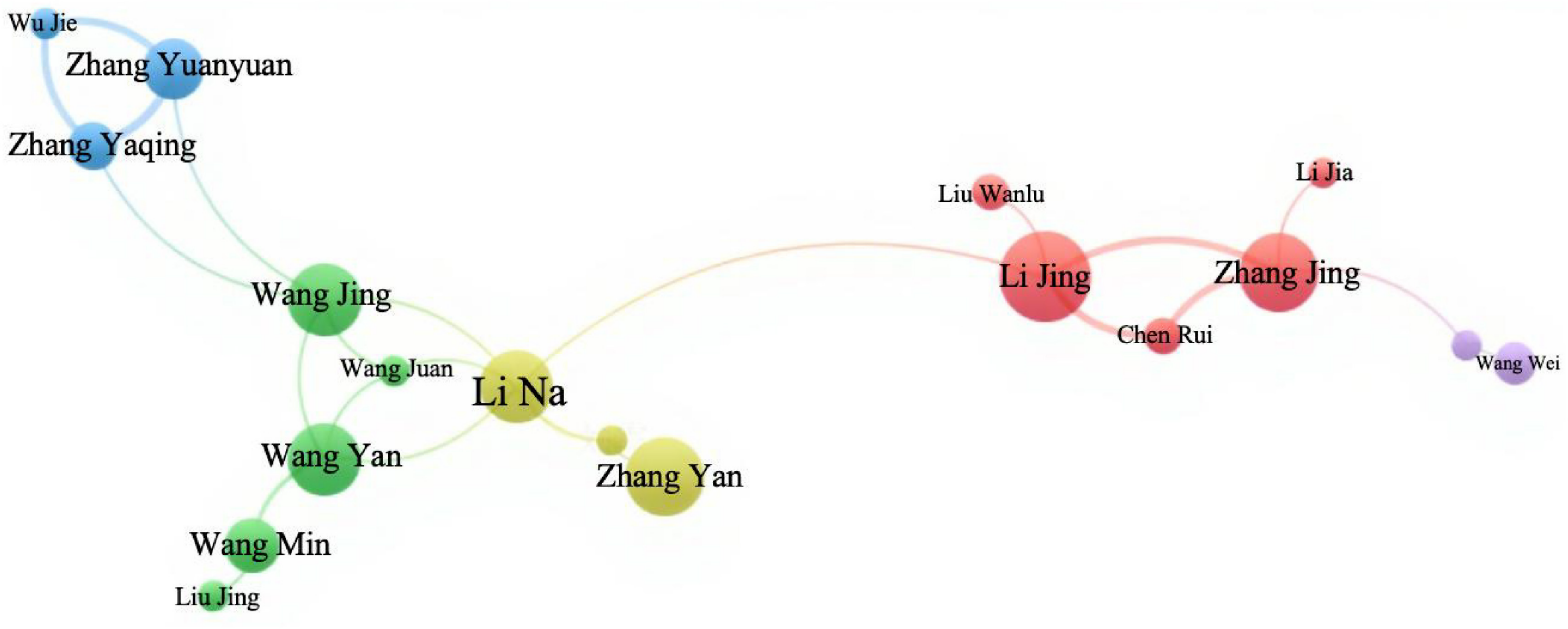
Collaborative network map of Chinese authors.

According to Price’s law (15): M = 0.749 × (N_*max*_)1/2, it was calculated that authors with ≥ 4 publications were classified as high-yield international authors, while authors with ≥ 3 publications were high-yield Chinese authors. Table 4 list the top five authors by the quantity of Chinese and English publications. A total of 17,872 international authors published papers related to newly graduated nurses, of which 221 authors published ≥ 4 papers. According to Figure 3, the core author collaboration network in the field of newly graduated nurses abroad formed nine research clusters, the majority of which had two to three scholars. These clusters had strong intra-team collaborations but few academic interactions across clusters, and the only scholars with weak connections to other clusters were individuals with a limited number of nodes. In China, 7924 authors have published papers related to newly graduated nurses, of which 448 authors have published ≥ 3 papers. The collaborative network of core authors in the field of newly graduated nurses in China has formed six major research clusters, as shown in Figure 4. The size of each cluster varies significantly, with the largest cluster having five scholars and the other clusters having two to four members, which is typical of a miniaturized research team.

**TABLE 4:** Top five Chinese and international authors in terms of publications in the field of newly graduated nurses from 2015 to 2025.

Classification	Author	Number of publications	Total link strength
International	Mckenna, Lisa	21	24
	Leino-Kilpi, Helena	9	25
	Cope, Vicki	9	13
	Walker, Stacy E.	9	10
	Palese, Alvisa	9	6
Chinese	Li Jing	15	6
	Wang Lixiang	13	36
	Wu Xingjuan	13	10
	Zhang Jing	13	5
	Zhang Yan	13	0

### 3.5 Keyword co-occurrence

There were 244 nodes, 2083 links, and a density of 0.0703 for the English keywords of new studies about nurses, and 270 nodes, 1554 links, and a density of 0.0428 for the Chinese keywords. Table 5 displays the top 10 high-frequency terms in both Chinese and English.

**TABLE 5:** Top 10 high-frequency keywords in the field of newly graduated nurses from 2015 to 2025.

Database	Keywords	Centrality	Frequency
WOS	Care	0.03	437
	Impact	0.06	320
	Experiences	0.04	317
	Transition	0.02	316
	Education	0.03	315
	Nurses	0.05	289
	Perceptions	0.07	257
	Health	0.02	222
	Nursing education	0.03	221
	Nursing students	0.03	168
CNKI	Newly graduated nurses	0.51	736
	Nurses	0.35	352
	Training	0.19	290
	Pre-service training	0.06	149
	Operating room	0.08	147
	In-service training	0.05	117
	Nursing management	0.10	112
	Influencing factors	0.07	110
	Qualitative research	0.10	100
	Transformation shock	0.04	100

### 3.6 Keyword clustering

**FIGURE 5:**
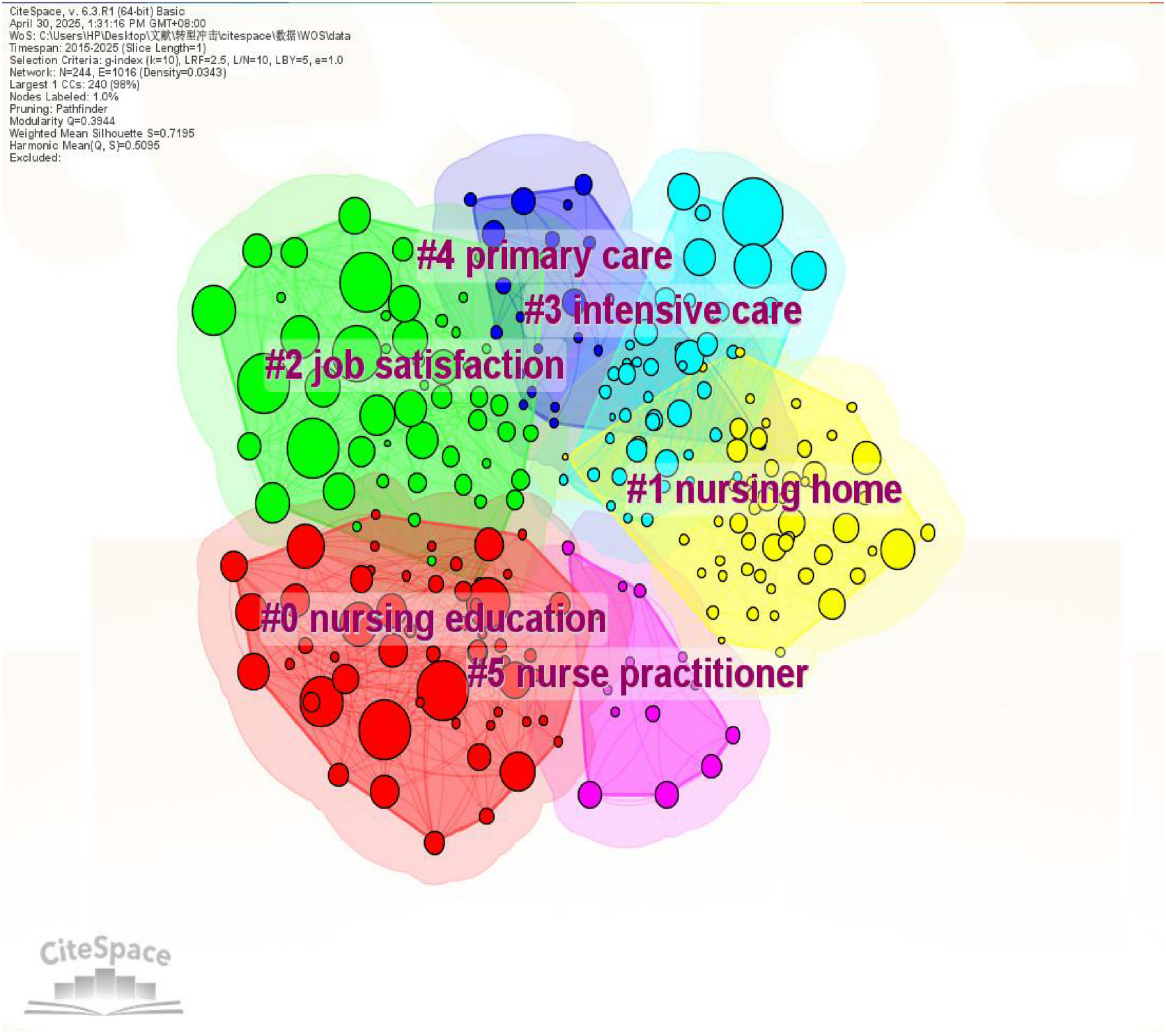
Clustering of keywords in English literature on new nurse research.

**FIGURE 6:**
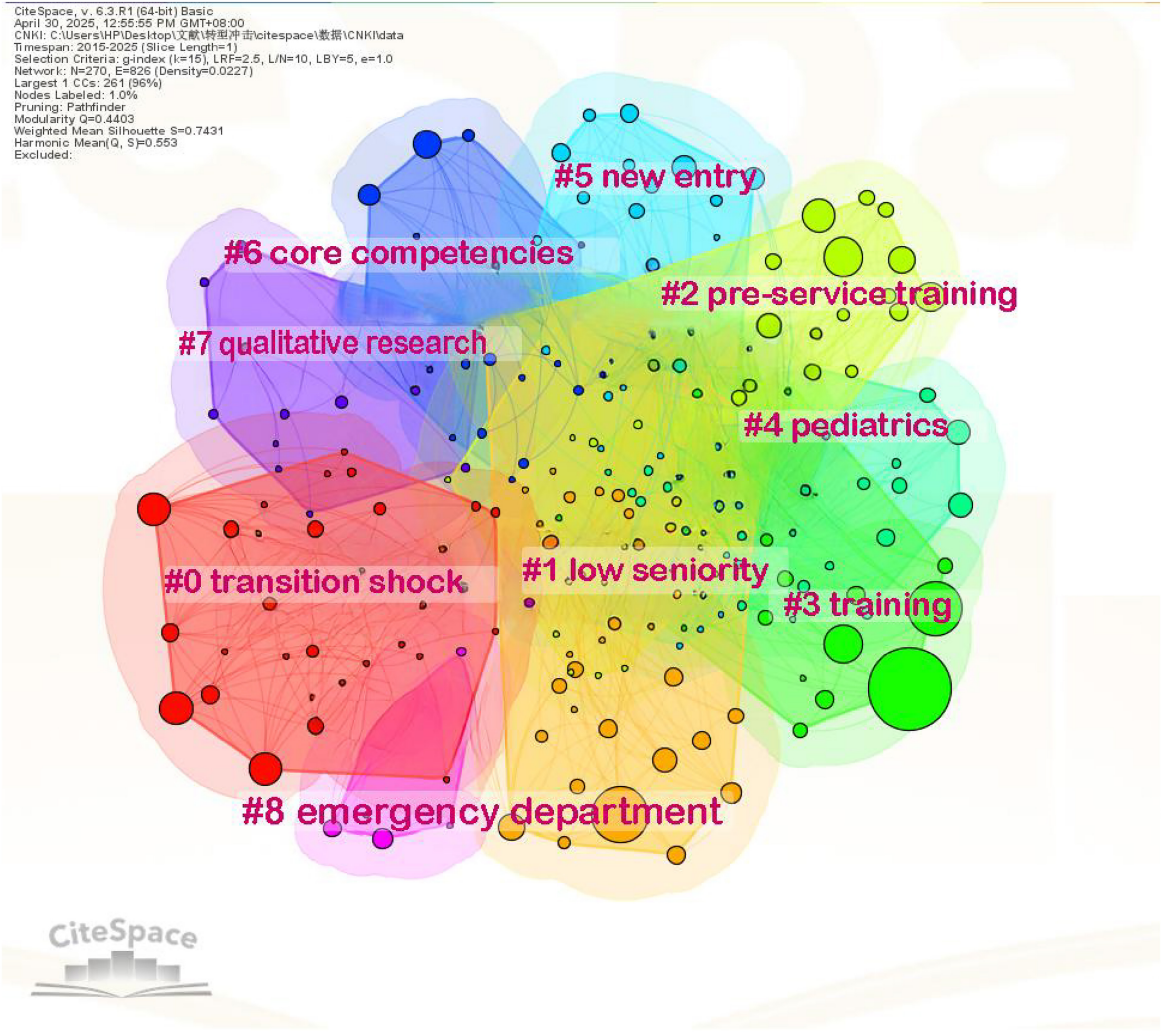
Clustering of keywords in Chinese literature on new nurse research.

The English literature clustering mapping of new nurse research has a module value of *Q* = 0.3944 and an average contour value of *S* = 0.7195, forming six keyword clusters, including nursing education, nursing home, job satisfaction, intensive care, primary care, nurse practitioner; Chinese literature clustering mapping *Q* = 0.4403, *S* = 0.7431, a total of 9 keyword clusters were formed, including transition shock, low seniority, pre-service training, training, pediatrics, new entry, core competencies, qualitative research, emergency department (Figures 5, 6).

### 3.7 Keyword burst

**FIGURE 7:**
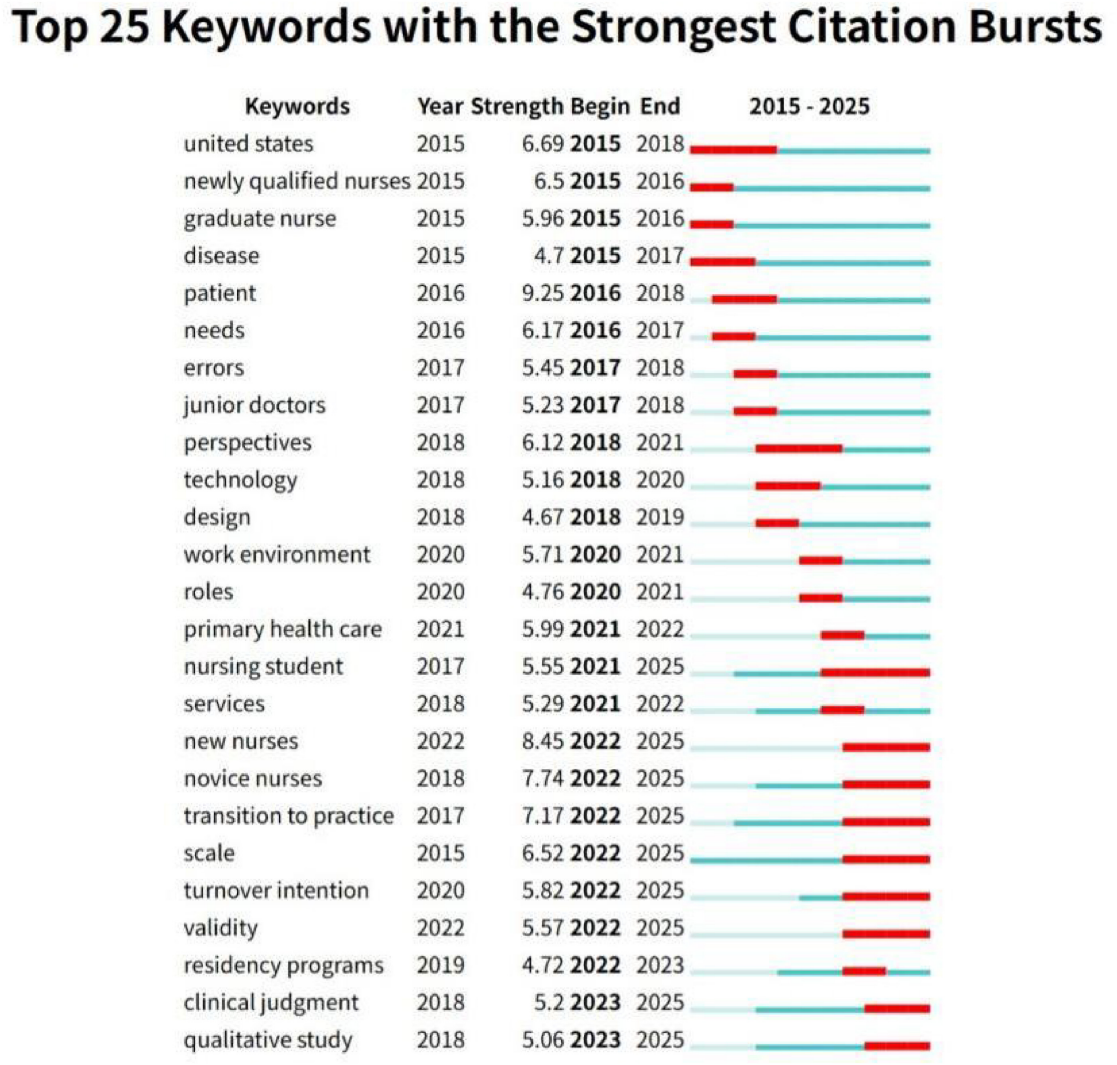
English keyword emergence map in the field of newly graduated nurses.

**FIGURE 8:**
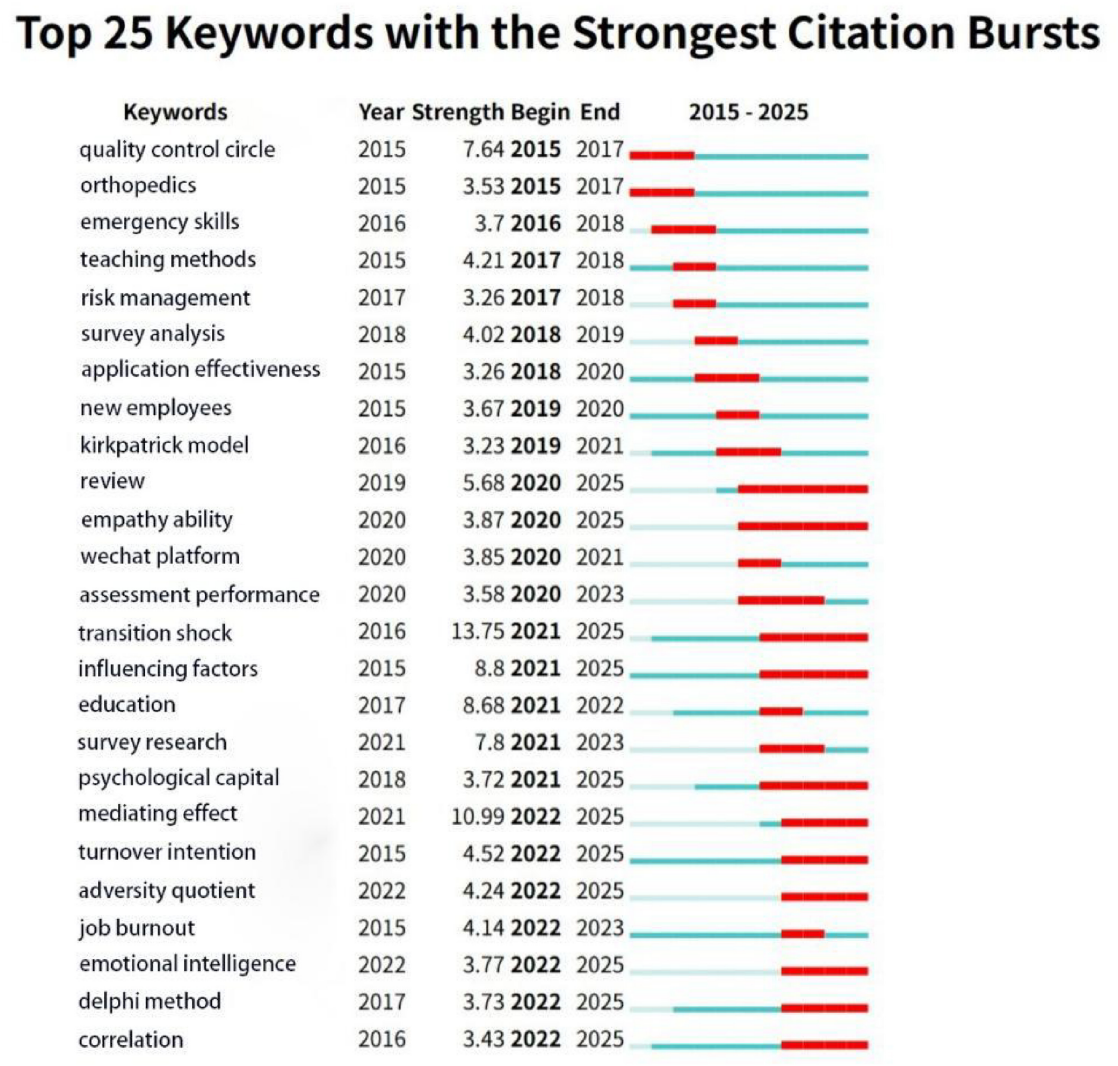
Chinese keyword emergence map in the field of newly graduated nurses.

Burst words are keywords that appear with a sudden increase in frequency during a certain period of time, and recent burst words can reflect the frontier of research to a certain extent (16). The top 25 outbreak keywords in Chinese and English for new nurse research are shown in Figures 7, 8.

In the English literature, the terms with the highest burst strength include “patient,” “newly graduated nurses,” “novice nurses” and “transition to practice.” The terms that have most recently become hotspots are “transition to practice” and “clinical judgment.” In the Chinese literature, the terms with the highest burst strength include “transition shock,” “mediating effect” and “quality control circle.” The terms that have most recently become hotspots are “transition shock,” “influencing factors,” and “Delphi method.”

## 4 Discussion

### 4.1 Current status of research in the field of newly graduated nurses

Through an analysis of the English and Chinese literature from 2015 to 2025, this study highlights geographical disparities and the global research landscape concerning newly graduated nurses. The English literature exhibits a consistent upward trend in terms of publications, particularly after 2020. This increase is noteworthy and could be attributed to the global community’s heightened awareness of the nursing human resource deficit. The COVID-19 pandemic exposed shortages and instability in the global nursing workforce, prompting increased research on newly graduated nurses’ occupational adaptation, mental health, and training systems (17, 18), with a view to cracking the structural contradiction between the distribution and demand of nursing human resources. Even though the number of publications in Chinese literature peaked between 2018 and 2020, it began to decline after 2021. This trend may be attributed to several key factors. Firstly, after 2020, Chinese nursing research priorities shifted significantly toward pandemic-related topics, diverting attention and resources away from traditional themes like new nurse training (19). Additionally, by 2020, China’s 2016 “Training Outline for Newly Entered Nurses” (10) had been widely adopted across healthcare institutions, reducing the demand for foundational studies on standardized training systems. Furthermore, some nursing journals expanded their coverage into specialized fields such as geriatric care and artificial intelligence in nursing, compressing publication space for new nurse topics (20). These developments collectively stand in contrast to sustained growth in English literature, ultimately explaining the divergence in publication trends between Chinese and international scholarship.

In terms of the distribution of countries and institutions, the United States is firmly in the first place with 36.43% of English-language literature, and its centrality index (0.40) indicates that it has significant influence in the global research network of newly graduated nurses, followed by developed countries such as Australia and the United Kingdom. While China ranks third in English output volume, its contribution percentage remains below 10%, suggesting room for greater international engagement. Notably, high-yielding institutions globally, such as Monash University and the University of Sydney, are concentrated in countries like Australia and the US. Within China, research output is primarily driven by major medical university hospitals.

In terms of journal distribution, international studies concentrate on professional journals like Nurse Education Today, which emphasize the fusion of educational theory and clinical practice; Chinese journals concentrate on nursing and management topics. This difference represents complementary perspectives rather than a hierarchy. The international focus provides robust models for education-practice integration, while the Chinese perspective offers valuable insights into structured training implementation and localized management challenges.

Author collaboration networks present the same models. The international landscape appears fragmented into numerous small clusters, indicating prolific but potentially isolated research pockets with limited cross-team synergy. In China, the collaboration pattern is similarly characterized by small, often institution-bound teams, with some authors showing minimal external links. This “siloed” approach, observed in various national contexts, poses a significant challenge globally. It limits the scope and depth of research, particularly for multifaceted issues requiring diverse expertise (21). Moving forward, both international and Chinese research communities stand to benefit immensely from strategically building larger, more interconnected, and interdisciplinary collaborative networks. Participation in international multi-center studies could systematically compare new nurse development pathways across cultures, enriching both local practices and global understanding.

### 4.2 Analysis of research hotspots related to newly graduated nurses

The results of keyword co-occurrence and clustering show that both Chinese and English literature on newly graduated nurses share common core issues, but also have different emphases due to differences in geographical practice contexts and research perspectives. The theme of “mental health” was emphasized in both Chinese and English literature. The English literature used concepts like “job satisfaction” and “impact” to examine the connection between newly graduated nurses’ career stability and mental health (22). While the Chinese literature reveals the formation mechanism of psychological stress during the transition period through themes like “influencing factors” and “qualitative research” (23). This commonality suggests that the mental health of newly graduated nurses is a common challenge in the global nursing field, and that cross-cultural comparative studies should be strengthened in the future to refine universal intervention strategies.

The high frequency keywords in the English literature focus on “nursing education,” “experiences,” and “transition,” emphasizing the transition from theoretical learning to clinical practice and the role of the education system in supporting the professional development of newly graduated nurses. International studies, exemplified by programs like the American Nurses Credentialing Center’s (ANCC) “Transition to Practice” model which provides structured 6–12 month residencies incorporating mentorship and simulation training (24), have optimized the clinical competence development of newly graduated nurses by exploring diverse educational modes, and focusing on their psychological adjustment and professional identity construction in the early stages of their entry into the profession (25, 26). In the meantime, the term clustering map of English literature included the newly popular idea of “nursing home.” The growth of newly graduated nurses’ professional competence is intimately linked to the enhancement of nursing quality in nursing homes, which are a significant provider of long-term care services (27). The turnover rate for newly graduated nurses in nursing homes is much greater than in normal hospitals because of the challenges of interdisciplinary teamwork, integrated management of patients with multiple diseases, and high-intensity care demands (28). Some western nations use economic incentives and policy action to maximize the distribution of human resources in order to address this issue. In Australia, for example, the government has launched the “Aged Care Workforce Strategy,” which enhances the sense of belonging of newly graduated nurses through salary subsidies, special continuing education funds, and career advancement pathways (29).

In 2016, China’s National Health Commission issued the “Training Outline for Newly Entered Nurses (Trial)” (10), which clearly stipulates that newly graduated nurses need to undergo 2 years of standardized training. Consequently, practical subjects like “pre-service training,” “nursing management,” and “qualitative research” are more prevalent in Chinese literature. Searching for the above keywords, it can be seen that the Chinese literature mostly discusses the standardized design of the training system (30). The keyword clustering results further show that Chinese studies focus on the direction of “transition impact,” “core competencies,” and “emergency department,” highlighting the shortcomings of newly graduated nurses’ competencies in specialized scenarios.

In terms of the complementarity between the Chinese and English studies, the intervention experience of the English literature on the “career development of newly graduated nurses in nursing homes” can provide a reference for the retention of talents in China’s primary healthcare institutions and long-term care facilities; the Chinese literature on the “development of competence in specialized scenarios” can complement the practice gap of international studies on the adaptation to complex clinical environments. In addition, future research needs to focus on the impact of digital transformation on the training of newly graduated nurses-such as the application of virtual simulation technology in training, the development of AI-assisted decision-making skills, and the comparison of the effects of training policies under different healthcare systems, so as to build a support system for newly graduated nurses that combines both local characteristics and international perspectives.

### 4.3 Analysis of research frontiers related to newly graduated nurses

Studies on newly graduated nurses, both in Chinese and international contexts from 2015 to 2020, commonly focused on foundational themes such as role transition, clinical adaptation, and standardized training. However, analysis of keyword bursts reveals a significant divergence in research frontiers post-2021.

Chinese research since 2021 demonstrates a deepening engagement with the psychological and systemic challenges of newly graduated nurses’ transition into practice. “Transition shock,” a key emergent concept throughout the Chinese literature over the previous 5 years, remained the focus of research. This suggests that the Chinese academic community has remained highly concerned about the psychological pressure, professional identity crisis, and lack of clinical practice ability of newly graduated nurses during the period of role transition from students to clinical workers (31, 32). This theme frequently co-occurs with keywords like “influencing factors” and “psychological capital” (33, 34), suggesting that rather than focusing only on describing the phenomena, future research will focus on applying psychological theories to explore the underlying mechanisms of newly graduated nurses’ adaptation. Meanwhile, the rise of terms like “Delphi method” indicates that future studies should develop systematic occupational adaptation support strategies by combining individual needs and evidence-based practice.

Conversely, the sustained emergence of “transition to practice,” “clinical judgment” and “turnover intention” in the English literature reflects international scholars’ exploration of career transition support, clinical decision-making thinking, and retention strategies for newly graduated nurses (35, 36). Furthermore, the emergence of the term “nursing student” suggests a shift in the perspective of research. The overlap of this concept with “transition to practice” on the timeline suggests that the causal relationship of “education to practice” is being explored in depth in the international community. This suggests that in the future, we can track the formation of nursing students’ professional identity, the effects of educational interventions, and early stressors, rather than only focusing on post-entry problems. This shift in the center of gravity of the study reflects a prospective concern for the competency gap of newly graduated nurses and also promotes a paradigm shift from “practice response” to “education and prevention” in the academic community.

Differences in the research frontiers concerning newly graduated nurses between China and the international contexts reflect distinct cultural perspectives and, more importantly, embody significant opportunities for mutual learning. Currently, Chinese research is delving deeply into the psychological mechanisms of newly graduated nurses’ transition adaptation and actively developing scientific support systems. In contrast, international research places greater emphasis on cultivating newly graduated nurses’ clinical judgment, exploring retention strategies, and increasingly recognizes the role of nursing education in facilitating professional adaptation. These differences are not barriers; rather, they constitute a complementary body of knowledge. The international focus on developing clinical judgment and early intervention strategies for nursing students offers flexible models that can be adapted for various contexts, including China. Simultaneously, the Chinese research focus on dissecting the complex psychological mechanisms of transition adaptation and exploring localized support systems contributes valuable experiences for addressing adaptation challenges in diverse settings.

These distinct research perspectives will inject new vitality and ideas into the development of global support strategies for newly graduated nurses. Key priorities for future research globally include: (1) Integrate psychological insights with practical interventions, specifically utilizing research findings on transition shock and psychological capital to optimize the design of transition support programs and clinical training plans for newly graduated nurses; (2) Shift the intervention perspective earlier, integrating competencies like professional identity formation and early stress management into nursing curricula to better prepare graduates; and (3) Fostering collaborative cross-cultural research to evaluate and synthesize diverse approaches, such as combining international competency frameworks with context-specific, locally adapted methodologies to optimize new nurse support systems globally.

## 5 Conclusion

In conclusion, this study systematically mapped the current status, hotspots, and frontiers of research on newly graduated nurses from 2015 to 2025 by visualizing both the Chinese- and English-language literature. According to the state of study, cross-team cooperation remains limited both within Chinese research communities and internationally. English-language literature continues to expand, whereas Chinese-language literature shows a gradual decline. Chinese and English research hotspots center on pre-service training and the development of specialist competence, while English focuses on nursing education and professional experience. Both Chinese and English address the mental health and training of newly graduated nurses. In terms of research frontiers, the English literature highlights the direction of “clinical decision-making ability” and “pre-professional intervention,” while the Chinese literature continues to deepen the mechanism of “transition impact” and the exploration of localized support systems. In the future, we must advance the deep integration of education and practice, optimize the digital transformation path of new nurse training, fortify interdisciplinary collaboration and international exchange, and create a support system for newly graduated nurses’ professional development that considers both local and global perspectives.
